# Physical properties of porphyrin-based crystalline metal‒organic frameworks

**DOI:** 10.1038/s42004-021-00484-4

**Published:** 2021-04-01

**Authors:** Sreehari Surendran Rajasree, Xinlin Li, Pravas Deria

**Affiliations:** Department of Chemistry and Biochemistry, Southern Illinois University, Carbondale, IL USA

**Keywords:** Metal-organic frameworks, Energy, Electrochemistry, Porous materials, Photochemistry

## Abstract

Metal**‒**organic frameworks (MOFs) are widely studied molecular assemblies that have demonstrated promise for a range of potential applications. Given the unique and well-established photophysical and electrochemical properties of porphyrins, porphyrin-based MOFs are emerging as promising candidates for energy harvesting and conversion applications. Here we discuss the physical properties of porphyrin-based MOFs, highlighting the evolution of various optical and electronic features as a function of their modular framework structures and compositional variations.

## Abstract (web summary)

Porphyrins have been incorporated into metal–organic frameworks in a periodic fashion in order to exploit their unique photophysical and electrochemical properties. This article reviews progress in the field, focusing on the fundamental physical properties that arise in porphyrin-based MOFs.

## Introduction

Metal**‒**organic frameworks (MOFs) are molecular assemblies^1–4^ that have attracted significant interest, mainly for their high specific porosity (reaching ~8000 m^2^ g^−1^)^5^. These frameworks are constructed by interconnecting organic linkers or struts with metal nodes or secondary building units (SBUs) through metal–carboxylate or metal–nitrogen (e.g., ligands with pyridine, imidazole moieties) coordination bonds. While these frameworks can be built in both crystalline and amorphous forms, the former (sometimes with a controlled degree of missing components or defects) have gained more attention over the past two decades. The versatile directionalities and connectivities of SBUs with linkers have led to enormous topological possibility and modularity. These features have been further expanded via reticular chemistry, generating many homologs or isoreticular structures simply by replacing the strut or node with another of comparable symmetry, allowing for the inclusion of a wide range of tailored functionalities. During their early development, MOFs were found to be attractive for the storage of low-density gases (e.g., hydrogen and methane) as transportable fuel owing to their high gravimetric surface areas^6–11^; targeting the optimal volumetric surface area is still an ongoing development^5^. Since the building blocks are precisely fixed in ordered arrays at their topology-defined positions in these polymeric frameworks, numerous chemical, electrochemical, photochemical, and photoelectrochemical developments have surged in the past decade^12–16^. The design flexibility of MOF-based systems was additionally widened via numerous post-synthetic approaches^17–22^. Therefore, MOFs provide a great platform where the electronic symmetry of the linkers and the topology-defined three-dimensional (3D) networks can manifest modular yet transformative physical properties^23–27^.

**Fig. 1:**
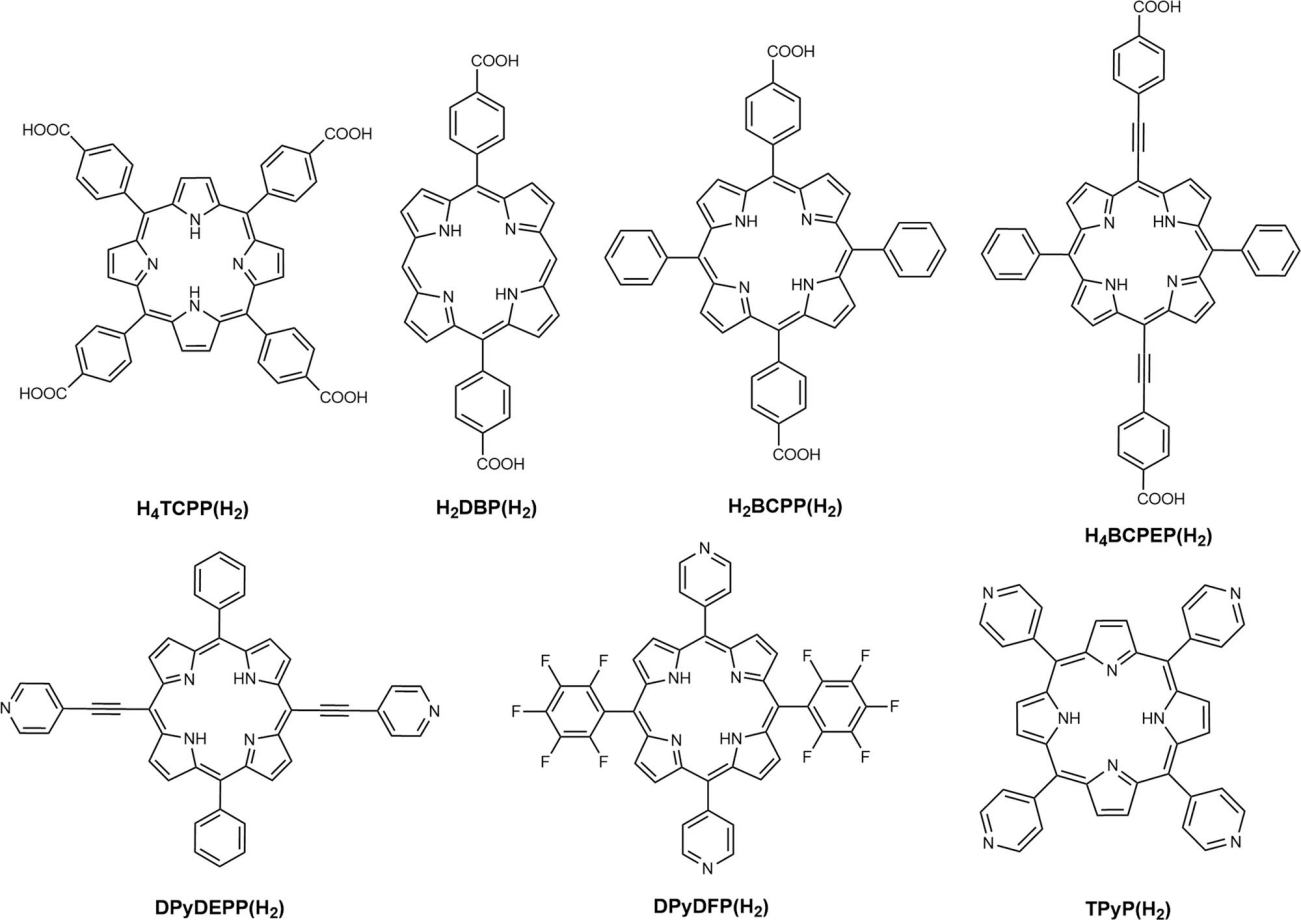
Porphyrin-derived MOF linkers. Examples of carboxy- and pyridyl-porphyrin linkers used for the construction of MOFs that are exploited for photophysical, photochemical, electrochemical, and photoelectrochemical developments.

The design of a MOF for a specific desired application starts with a careful choice of its linker based on its symmetry that is defined by its aromatic core and number (counted as topicity) and length of coordinating ligand arms. From this consideration, highly symmetric planar aromatic linkers are commonly used in MOF synthesis; in this regard, the porphyrin macrocycle provides a perfect *D*_4*h*_ core. The most basic and widely used porphyrin linkers are *meso*-substituted tetra-carboxyphenyl and 4-pyridyl linkers (Fig. 1), which have generated a plethora of MOFs^16,27–31^.

The uniqueness of porphyrin-based MOFs also comes with decade-old rich chemistry and optoelectronic features. For example, porphyrins are one of the most intense light absorbers with the Soret band (at 400–440 nm) absorptivity being as high as 10^5^ cm^−1^ M^−1^, with relatively weak lowest-energy *Q*-band transitions. However, these optical properties (e.g., transition energy, oscillator strength, lifetimes, and so on) can be synthetically tuned^32,33^ by altering the electronic symmetry (symmetry of the *π*-conjugated core defining the frontier molecular orbitals) through the extension of the conjugation along the molecular axis; i.e., by differentiating the *x*-polarized transition dipole from its degenerate *y*-polarized one (as seen in the common *D*_4*h*_ cores). Besides, porphyrins are probably the most versatile and modular molecular systems as (i) all four *meso*- and eight *β* positions can be functionalized, and (ii) they accommodate a wide range of metal complexes through tetra-pyrrole-based ligation. Therefore, by altering the side group functionality and the central metal identity, the electronic properties can be tuned. Axial coordination can further play a pivotal role to define the electronic structure and functions of the system. Thus, metalloporphyrins are chosen by the natural evolution process as a range of key functional cofactors, like oxygen-binding heme, oxygen-activating *cyt*-P450 (and peroxidase), and various *cyt*-based redox shuttles, where the different activities are achieved by tuning the side groups and choosing the appropriate axial coordination^34–37^. Likewise, the highly absorptive chlorophylls that are found in the green plant or purple bacteria are based on a porphyrinoid core, where a lower electronic symmetry is exploited to improve the *Q*_*x*_-derived oscillator strength. The design criteria for molecular and supramolecular porphyrin-based high absorptive visible–near infrared (Vis-NIR) chromophores are well established. On the other hand, design principles to achieve such properties in solid porphyrin assemblies, i.e., within MOFs, are an ongoing development as solvothermal assemblies of synthetically accessible porphyrins can provide scalable framework compositions with new properties. Some commonly used porphyrin-based linkers are shown in Fig. 1. Porphyrin-based crystalline frameworks have been known for about 30 years or so^38,39^; works by Zubieta as well as Robson showcased some of the first porphyrin-based 3D frameworks. However, systematic development of porphyrin MOFs started in 2006^40^. The pillar-paddlewheel MOFs, which appeared first in 2009, are mixed linker systems, where 5,15-dipyridyl porphyrin derivatives act as pillars for tetracarboxy linkers forming the paddlewheels^41^. Alternatively, tetra-carboxyporphyrin-based paddlewheel layers were successfully stacked in an AA or AB fashion with 4,4’-dipyridyl or 1,4-diazabicyclo[2.2.2]octane (DABCO) pillars^42,43^. Initial developments were aimed to explore novel structures harnessing metalloporphyrin-based chemical catalysis that takes advantage of the high local concentration of both the catalysts and substrates within the pores^44^.

**Fig. 2:**
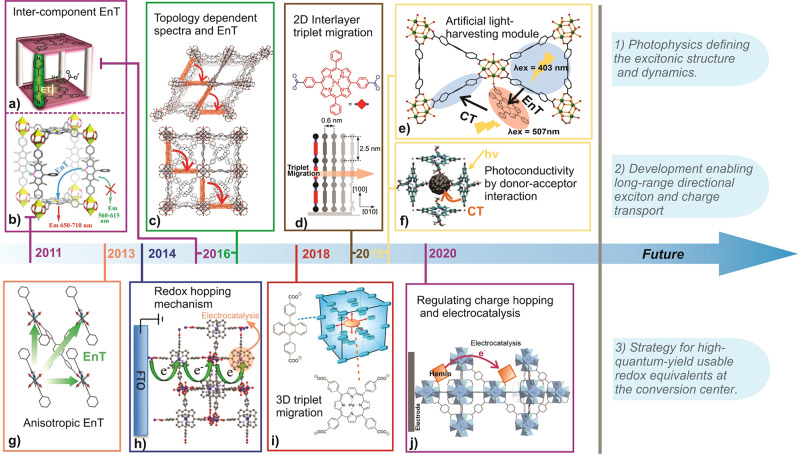
Chronology summarizing the photophysical and electrochemical developments of porphyrin MOFs. The present article describes the photophysical [**a**^58^; **b**^56^; **c**^59^; **d**^45^; **e**^71^; **f**^88^; **g**^57^—including energy transfer (EnT) and charge transfer (CT) processes] and electrochemical [**h**^81^; **i**^67^; **j**^84^] developments along with potential future directions. The figure panels were reproduced from corresponding literature with permission from the American Chemical Society. (**a** reprinted with permission from ref. ^58^; Copyright (2016) American Chemical Society. **b** reprinted with permission from ref. ^56^; Copyright (2011) American Chemical Society. **d** reprinted with permission from ref. ^45^; Copyright (2019) American Chemical Society. **h** reprinted with permission from ref. ^81^; Copyright (2014) American Chemical Society. **i** reprinted with permission from ref. ^67^; Copyright (2018) American Chemical Society.).

Photophysical, photochemical, electrochemical, and photoelectrochemical developments with porphyrin MOFs are relatively recent and started with observations of strut-to-strut energy and charge transfer. Figure 2 highlights a chronology of major photophysical and electrochemical developments. Early studies involved Zn^II^-polyhedra, particularly the paddlewheel SBU, which are now commonly used to delineate surface-anchored layered MOF known as SURMOFs^42,45^. Later developments commonly exploited robust Zr^IV^-oxo-based frameworks, where the strong Zr-carboxylate bond exerts hydrolytic and mechanical stability—lessons learned from the UiO-66 class of frameworks^46^. The impressive stability of Zr-carboxylate MOFs in a wide range of pH (1–10) is expected to play a pivotal role in many photocatalytic and photoelectrocatalytic developments. However, these frameworks are not stable in neutral phosphate buffer or basic media. Leading in this class, Zhou’s group developed a series of porphyrin-based MOFs exploiting the symmetric 5,10,15,20-tetra-(4-carboxyphenyl)porphyrin (H_4_TCPP(M)), a tetra-topic porphyrin with varying central metal ions—these MOFs are named as porous coordination networks, e.g., PCN-222(M) (M = H_2_, Mn, Fe, Co, Ni, Cu, Zn)^31^. These TCPP(M)-based MOFs were realized by varying the synthetic condition (like linker-to-metal ratio, pH, concentration, and most importantly the temperature) to produce either kinetic or thermodynamic products^47^.

Topology-dependent positioning of the porphyrin linkers offers a unique opportunity to modulate and achieve unusual optoelectronic properties, something that is only seen in biological light-harvesting systems. Realizing such properties has been synthetically challenging in other supramolecular and macromolecular architectures. For the framework-organized porphyrins, the pore geometry dictates the electronic interaction in the ground and excited states, whereas the pore channels facilitate dielectric modulation, including small molecule diffusion. Porphyrin-based MOFs have been well studied in the past decades and may be broadly classified into three categories: (1) mixed-linker systems—commonly used to probe intercomponent energy transfer (EnT), (2) pure-linker systems—commonly used to probe topology-dependent excitonic and electronic properties, and (3) low-density linker/ligand systems—mostly used for charge separation and transport. The low-density compositions can be of two types, where the porphyrins are (a) scrambled but essential structural components and (b) non-essential post-synthetically incorporated ligands within the framework.

Review articles on porphyrin-based MOFs (as well as general MOF articles) can be found in literature discussing features, such as synthesis, stability, porosity (gas adsorption, separation, and storage), film growth, and catalytic studies^12,48–51^. Here, we focus on the underlying fundamental physical properties and strategies to regulate energy and charge transfer and transport processes, as these understandings will facilitate future materials developments. Impressive progress has been made with porphyrin MOFs, providing us with a glimpse of what is possible toward building light-harvesting antenna that manipulates various (directional) EnT processes in singlet and triplet manifolds (Fig. 2a–d, g, i): constructing artificial light-harvesting modules (Fig. 2e), exploring charge transport behavior in MOFs (Fig. 2f, h), and leading to modular electrocatalytic developments (Fig. 2j).

## Photophysical properties and excited-state processes in porphyrin-based MOFs

With their extensive chemical and structural tunability and wide range of pore geometries, it is expected that the interchromophoric interactions in porphyrin-based MOFs can be tuned to achieve unique excited-state properties to define a plethora of photo-driven processes; these include the evolution of new spectroscopic features and excited-state dynamics (including photo-induced charge-carrier generation and their dynamics) that are different than the solution-dissolved molecular or macromolecular porphyrin-based compounds. The advent of various synthetic (de novo as well as post-synthesis) methodologies for the construction of porphyrin MOFs, experiments to probe EnT, in both singlet and triplet manifolds, as well as other photoinduced processes were explored by (1) varying the electronic properties of the porphyrin linkers, (2) modulating the extent of electronic coupling of porphyrins and other redox-active linkers, and (3) adopting state-of-the-art spectroscopic techniques to establish mechanistic understanding (Fig. 2).

### Singlet EnT

Molecular excitons (electron–hole pairs with sizable binding energy) in porphyrin-based MOFs can efficiently migrate to new sites before recombining via radiative and/or non-radiative processes including exciton splitting into charge carriers. In pure-linker systems, exciton migration involves a self-exchange-type hopping process, which has been described via the Förster or Dexter mechanism depending on the spin manifold of the excited population. Even though singlet excitons are short-lived, the Förster resonance energy transfer (FRET) process is highly efficient (seen in natural light-harvesting complexes in photosystem I/II) to facilitate a long-range exciton migration (i.e. >50 nm). The FRET rate constant (*k*_FRET_ = 2*πJ*²Θ/$\hbar$) depends on the interchromophoric electronic coupling *J* (eV), and the overlap integral Θ (eV^−1^) between the area-normalized donor fluorescence and acceptor absorption bands^52–55^. Here, *J* can be modulated by tuning the MOF topology, which defines the relative position and orientation of the chromophores, and Θ can be primarily tuned by the design of the individual chromophore linkers. In contrast, triplet excitons are long-lived, but the efficiency of the triplet–triplet energy transfer (TTET) process may be limited by the requirement of a shorter distance.

**Fig. 3:**
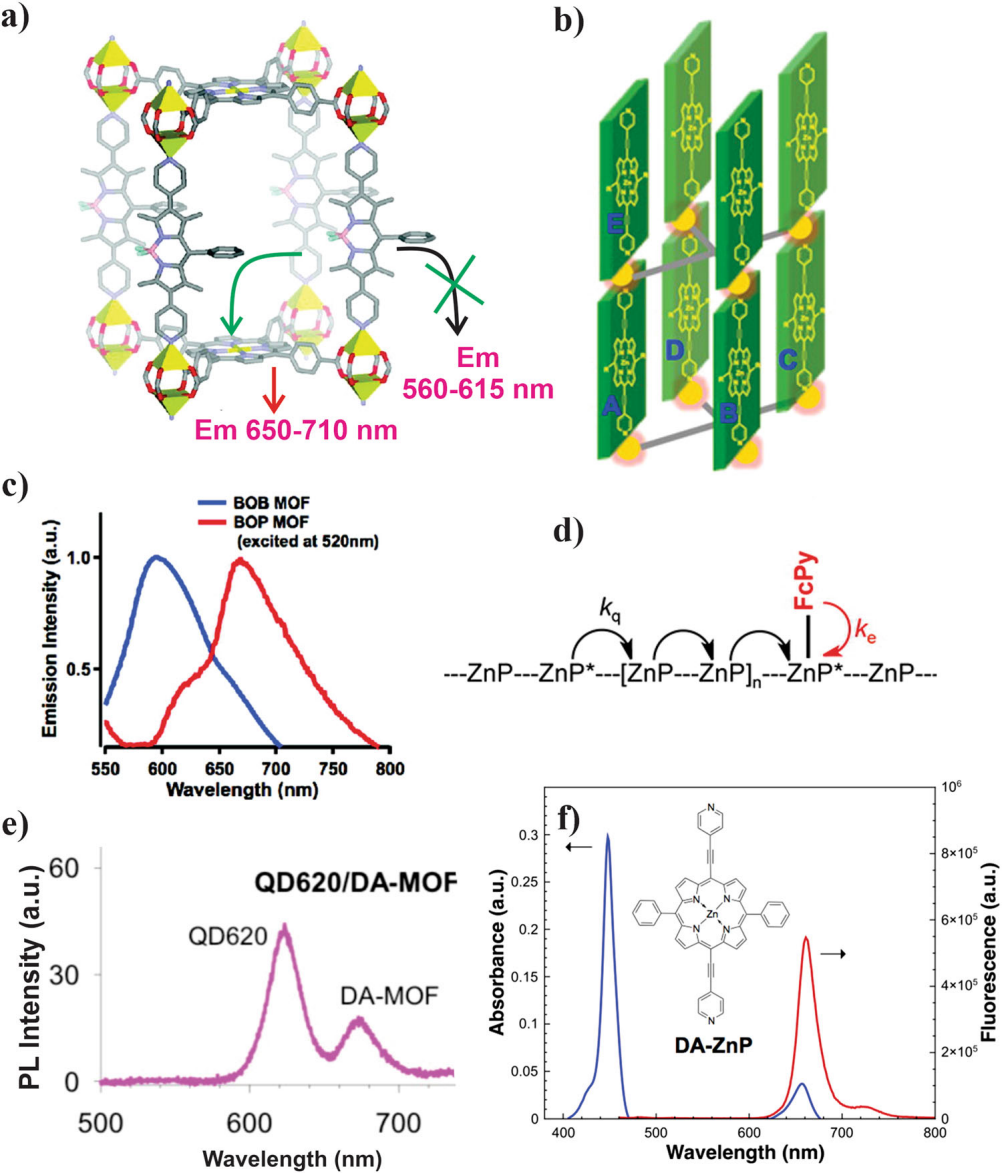
Intercomponent energy transfer in BOP MOF and DA MOF. **a** Energy transfer in BOP MOF; **b** cartoon representation of DA-MOF representing various crystallographic directions for exciton migration from A (to B–E directions); **c** emission spectra of BOB and BOP MOFs (*λ*_ex_ = 520 nm); **d** schematic representation of exciton migration and subsequent quenching processes adopted in amplified quenching experiments; **e** emission spectra of QD620@DA-MOF composites (*λ*_ex_ = 400 nm); **f** absorption (blue) and emission (red) spectra of DA-MOF highlighting a large Θ. **a**, **c** reprinted with permission from ref. ^56^; Copyright (2011) American Chemical Society. **b**, **d**, **f** reprinted with permission from ref. ^57^; Copyright (2013) American Chemical Society. **e** reprinted with permission from ref. ^55^; Copyright (2013) American Chemical Society.

One of the earlier strut-to-strut EnTs within porphyrin-based MOFs was reported by the Hupp group using a mixed-linker system. A zinc pillar paddlewheel MOF, named BOP (Fig. 3a), was prepared from H_4_TCPP(H_2_) and dipyridylbodipy linkers and a zinc(II) salt^56^. Given that bodipy linker absorbs light complementary to that of the TCPP(Zn) (obtained via an in situ metallation that occurred during the MOF synthesis), the BOP MOF was designed to cover a wide range of the visible spectrum. With a large spectral overlap between the emission of the dipyridylbodipy and the absorption of the TCPP(Zn), the BOP MOF was expected to show strut-to-strut EnT. For this reason, excitation of the bodipy component at 520 nm did not produce its characteristic emission at 590 nm; instead, an emission signal at 670 nm was observed from the TCPP(Zn) (Fig. 3c). The corresponding excitation spectra, for TCPP(Zn) emission probed at 670 nm, showed a bodipy contribution appearing at 520 nm. A control experiment with BOB MOF, where TCPP(Zn) was replaced with a tetratropic TCPB strut (TCPB = 1,2,4,5-tetrakis(4-carboxyphenyl)benzene with a large optical bandgap), showed a clear bodipy-based emission at 590 nm. This study exemplified the strut-A to strut-B EnT process within the crystalline MOF system.

With the appropriate alignment of the porphyrin linkers, Hupp and co-workers showed that the EnT efficiency can be a function of linker electronic symmetry achieved by altering the extent of *π*- conjugation. Two frameworks, F-MOF and DA-MOF, were constructed from dipyridylporphyrins pillars with varying conjugation length and Zn-TCPB paddlewheels^57^. The ethyne-elaborated dipyridyl linker DPyDEPP(Zn) (DPyDEPP(Zn) = [5,15-di(4-pyridylacetyl)-10,20-diphenyl]porphyrin), also denoted as DA-ZnP, possesses higher oscillator strength for its lowest energy *Q*_*x*_-derived transition that resulted in higher Θ (Fig. 3f) compared to the less conjugated DPyPFP(Zn) linker (DPyPFP(Zn) = [5,15-dipyridyl-10,20-bis(pentafluorophenyl)]porphyrin; Fig. 1). Exciton migration and the corresponding EnT processes were probed via an amplified quenching experiment using pyridyl ferrocene (FcPy) as the redox quencher installed at the zinc sites of the porphyrins. The respective emission intensities, analyzed by a Stern–Volmer approach against the FcPy/porphyrin ratio, provided exciton hopping matrices (Fig. 3d): in DA-MOF, a photogenerated exciton can visit ca 90 porphyrin struts (i.e., absolute displacement ~45 struts) within its lifetime. From the calculated electronic coupling constants along the different crystallographic directions, the anisotropic exciton migration was established. This feature was found to be topology dependent—defined by the shortest (adjacent) inter-porphyrin distance. The most probable (~55%) exciton hopping was estimated along the AB (Fig. 3b) direction with a ∼38 nm displacement, where the longest possible displacement (~58 nm) along the AE direction has only ∼21% probability. This pioneering study showed that an efficient directional EnT is possible in molecular assembly out of the protein-bound natural light-harvesting antenna system of PS-I/II.

Considering tremendous advancement in nanostructured optoelectronic materials, particularly metal–chalcogenides, novel hybrids involving MOF have been developed for light harvesting. These nanostructures, such as CdSe- and ZnS-based quantum dots (QDs), are commercially accessible and possess a broad phonon-coupled absorption and narrow emission bands. Although the specific absorptivities of such QDs are not as high as the molecular assemblies (of the same molecular mass), their synthetically accessible size-dependent electronic transitions are attractive and can be exploited to tune the complimentary absorption (achieving better coverage of the solar spectrum) and the emission bands primed for EnT to the MOFs. Hupp’s group prepared QD-MOFs hybrids based on their DA-MOF and F-MOF^55^. As discussed above, due to an increased conjugation the Soret band of the DPyDEPP(Zn) is redshifted and thus excitation at 400 nm does not produce any sizable porphyrin-based emission. However, a QD620/DA-MOF hybrid exhibited porphyrin emission at 680 nm (Fig. 3e) upon QD excitation at 400 nm. In contrast, QD550/DA-MOF hybrid exhibited a poor porphyrin emission as the 620 nm emission profile of the QD620 seemed to provide a better Θ (than the QD550 emission) to transfer its excited-state energy via *Q*-band excitation of the DA-MOF. The QD620/DA-MOF composite displayed photon harvesting at a wavelength where MOF does not absorb with a high (∼84%) EnT efficiency. It is important to note that the chromophoric struts, as well as the pore-incorporated chromophores or nanostructures, exist in an isolated manner avoiding aggregation and related unuseful (e.g., thermal) excited-state decay processes. The advantageous site isolation strategy was specifically demonstrated by Shustova and co-workers by probing the EnT process in a porphyrin MOF mimicking the green fluorescent protein (GFP)-cytochrome b_562_^58^. As such, the 4-hydroxybenzylidene imidazolinone (HB1) chromophore that is commonly found in the *β*-barrel capsule of GFP become non-emissive when extracted out of the native environment. Interestingly, when installed inside the Zn-porphyrin MOF scaffold, the emissive nature of HB1 chromophore was restored with a highly efficient FRET between HB1 and porphyrin linkers in the MOF. Diffusive (noncoordinating) encapsulation of HB1 was found to facilitate an enhanced orbital overlap between HB1 (donor) and porphyrin (acceptor) resulting a high (72%) EnT efficiency.

Since the interchromophoric distance impacts the *J*, FRET process over the adjacent porphyrin layers can be modulated by altering the interlayer distance, which can be achieved via collapsing a 3D pillared paddlewheel framework to a two-dimensional network or adjusting the height of the pillar. For such a study, Goswami et al. constructed TCPP(Zn)-based SURMOF films via a layer-by-layer approach^42,43^. Here, the zinc-paddlewheel layers of TCPP(Zn) linkers are arranged in a parallel face-to-face orientation held by bipyridine pillars with an interplanar distance of 14.7 Å. Replacement of the bipyridine pillars with monodentate pyridine via post-synthesis solvent-assisted linker exchange reaction resulted in a collapsed structure with a reduced interlayer distance of 6.9 Å and skewed orientation in a way that the TCPP–TCPP distance in the adjacent and alternate layers became 14.3 and 16.8 Å, respectively. Since this collapse did not lead to a significant change in distance between the closest transition dipoles (14.7 vs 14.3 Å), the spectroscopic parameters (such as excited-state lifetime) remained more or less unchanged. Using TCPP(Pd) zinc-paddlewheel as the final layer to terminate the exciton migration, the authors showed that the excitons can migrate ca 9 and 11 layers in pristine bipyridine 3D MOF and collapsed MOF, respectively. Interestingly, a relevant SURMOF with shorter DABCO pillars displayed an exciton migration length of 26 layers owing to a shorter strut–strut (as well as interlayer) distance (9.9 Å). This investigation confirmed that the distance between adjacent transition dipoles is more important for exciton migration than an average interlayer distance.

**Fig. 4:**
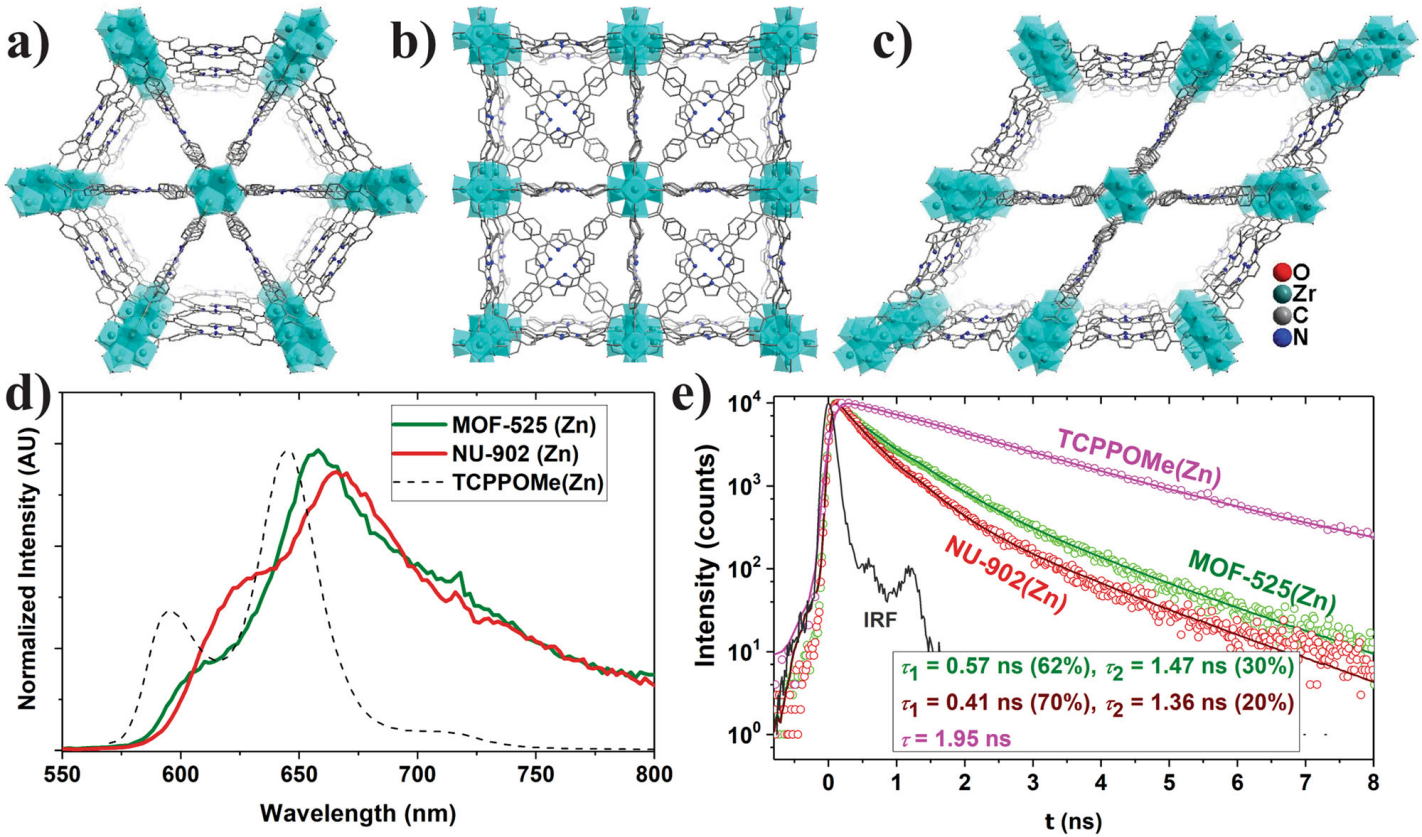
Topologically different TCPP MOFs and evolution of their photophysical properties. Crystal structure of **a** PCN-223, **b** MOF-525, and **c** NU-902; **d** steady-state emission spectra; and **e** transient emission decay profiles for MOF-525(Zn) and NU-902(Zn) relative to dissolved TCPP(Zn) in DMF solvent. Data presented in **d**, **e** were published^59^.

As mentioned earlier, topology defines the pore geometries within MOFs and therefore it dictates the interchromophoric distance and orientation. Structurally different TCPP-based Zr^IV^-oxo MOFs, prepared by varying synthetic conditions, were expected to offer ways to tune the transition–dipole interaction. Deria et al. studied the varying degree of exciton coupling among the TCPP struts by probing the evolution of the emissive properties within a series of topologically different zirconium-based porphyrin MOFs^59^. The electronic transition energies, obtained from the steady-state emission spectra, suggest that a lower optical band gap can be obtained as a result of stronger inter-porphyrin interaction with shorter interchromophoric distance (Fig. 4d). Supporting transient emissive decay profiles indicate that a stronger interchromophoric interaction that led to a lower optical bandgap also manifests a faster population decay (Fig. 4e). The transient emission spectral envelopes (Fig. 4d) maintained a broad feature similar to the corresponding steady-state spectra, which was attributed to the excitonic nature of the excited states in these solid porphyrin assemblies—also observed in pyrene-based MOFs^27^. Amplified emission quenching experiments with ferrocene-installed samples revealed a highly efficient singlet EnT process (with absolute displacement estimated to be ~57 in NU-902(Zn)), which is comparable to that determined for DA-MOF^57^.

Unlike other first-row transition metal ions, Zr^IV^ does not get inserted in the porphyrin macrocycle during a solvothermal MOF synthesis, which made possible preparation of a series of MOFs with free-base porphyrin linkers. Robust TCPP(H_2_)-based frameworks can thus serve as pH sensors exploiting the protonated linkers, TCPP(H_4_)^2+^, as an internal emission quencher^60^. Efficient EnT can lead to an amplified quenching to sense a significantly low concentration of protonated linkers as shown in TCPP(H_2_)-based Zr MOF PCN-223(H_2_) (Fig. 4a) by Morris and co-workers^61^. In these MOFs, the concentration of TCPP(H_4_)^2+^ moieties was varied by adjusting the pH of the solution. Protonation of pyrrole nitrogens in the porphyrins altered the electronic structure of TCPP(H_2_) in PCN-223(H_2_), which led to a luminescence quenching due to an efficient site-to-site EnT (*k* = 1.9 × 10^–12^ s^−1^ was obtained from Stern–Volmer analysis). Such amplified quenching process can be exploited in various Zr-TCPP(M) MOFs (e.g., PCN-221(H_2_)) as a fluorescence sensor to detect heavy metal with a lower limit of 0.01 µM for Hg^2+^ ions, where the trace amount of mercury ions—bound possibly via chemisorption through pyrrole nitrogens—act as emission quencher^62^.

### Triplet EnT

**Fig. 5:**
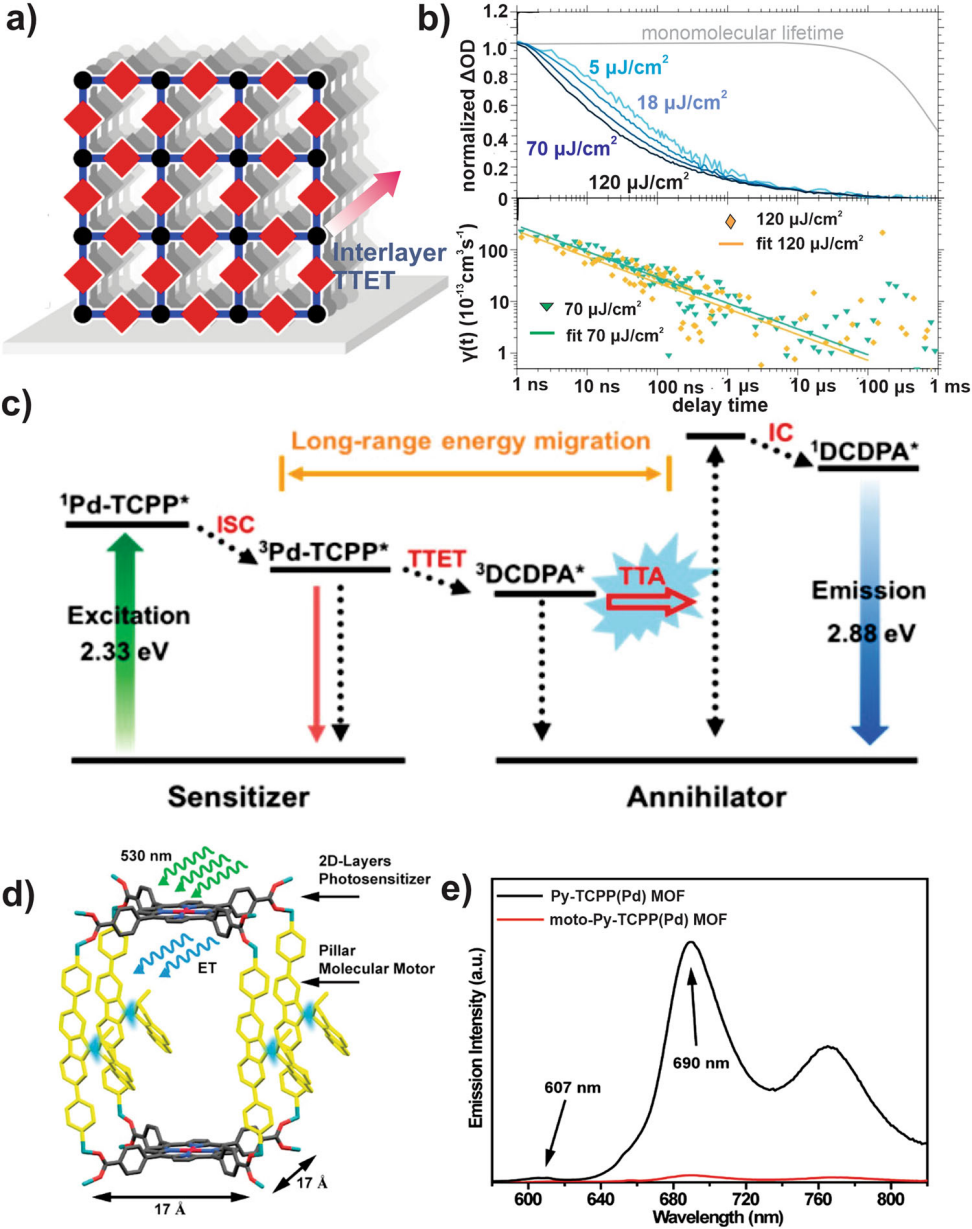
Exciton migration and other photophysical processes within triplet manifolds of various Pd-porphyrin MOFs. **a** TTET in BCPP(Pd) SURMOF with **b** normalized TAS kinetic traces collected at different excitation fluences (the monomolecular lifetime is shown for reference) and the corresponding TTA rate coefficient for the highest two excitation fluences (the solid line represents 1D diffusion model); **c** energy diagram showing TTET followed by TTA-UC process in TCPP(Pd)-DCDPA mixed-linker MOF; **d** schematic representation of EnT followed by a rotation of motor unit in moto-Py-TCPP(Pd) MOF; **e** comparison of emission spectra of Py-TCPP(Pd) MOF with moto-Py-TCPP(Pd) MOF. **a**, **b** reprinted with permission from ref. ^45^; Copyright (2019) American Chemical Society. **c** reprinted with permission from ref. ^67^; Copyright (2018) American Chemical Society. **d**, **e** reprinted with permission from ref. ^68^; Licensed by ACS, link: 10.1021/jacs.0c03063 and further permissions related to the material excerpted should be directed to the ACS.

Considering the reactive nature of the singlet oxygen (^1^O_2_), formed by quenching triplet excited states of common chromophores, natural photosystems adopted a preventive measure by efficiently quenching its triplet populations that may have formed within the light-harvesting pigment assemblies in ambient condition. However, artificial systems could be designed to perform under de-aerated environments, where the triplet excited states may be preferred for a series of desired photophysical developments as the long-lived triplet excitons could eventually drive photochemical transformations (which are commonly slower than the fast deactivation of the singlet excited population). Owing to their long lifetime, triplet excitons can be expected to migrate longer reaching distal sites, albeit short interchromophoric distance required for the double-exchange Dexter process. For efficient TTET, frameworks with closely positioned chromophores will be the primary choice. As seen above, SURMOFs potentially enable superior thin films of frameworks; in this regard, a different kind of SURMOF consisting of closely packed standing layers was developed by Wöll and Bräse and exploited for various spectroscopic investigations^32,33,63^. In one such study, Howard et al. probed TTET (Fig. 5a) in a palladium porphyrin-based SURMOF^45^ constructed from zinc-paddlewheel layers of BCPP(Pd) linker (BCPP(Pd) = 5,15-bis(4-carboxyphenyl)-10,20-diphenylporphyrinato)palladium(II)). The strong spin–orbit coupling ensured efficient triplet exciton generation via intersystem crossing (ISC) of the initially formed singlet populations. The limited rotational freedom for the *meso*-phenyl groups of the BCPP(Pd) linkers within SURMOF suppressed a wasteful thermal decay of the triplet populations and thus a longer triplet lifetime was achieved in such a stacked MOF structure compared to the unassembled solubilized H_4_BCPP(Pd) linker. The increased triplet lifetime was expected to bolster longer exciton migration in these solid frameworks. The formation and the dynamics of the triplet excitons were probed by varying pump fluence (Fig. 5b): the transient absorption spectra and corresponding kinetic data collected at higher pump fluence manifested a faster decay of triplet excitons via triplet–triplet annihilation (TTA). The *k*_TTET_ of 8 × 10^10^ s^−1^, recorded in this BCPP(Pd)-SURMOF, is far better than that can be achieved in disordered conjugated polymers^64^ and comparable to tetracene and pentacene crystals^65,66^.

In another study, TTET is observed in a TCPP(Pd)- and 4,4′-(9,10-anthracenediyl)dibenzoic acid (DCDPA)-based mixed-linker MOF, where the former linker acts as the photosensitizer and the latter one serves as an acceptor and annihilator—i.e., by efficiently transferring the ^3^TCPP(Pd)* energy to populate the T_1_ state of DCDPA^67^. Here, TTET followed by TTA resulted in a photon upconversion (UC) process within the MOF, which can be exploited in low-power in vivo imaging. In the framework, two ^3^DCDPA* linkers undergo TTA-UC to generate a high-energy ^1^DCDPA* (Fig. 5c), which subsequently relaxes to the ground state by emitting a photon of higher energy. In another recent work, TTET is observed in a TCPP(Pd) MOF constructed with Zn-pillar paddlewheel and bipyridine fluorene-derived acceptor as pillars (moto-Py-TCPP(Pd) MOF)^68^. EnT from ^3^TCPP(Pd)* to the pillar linkers was used to alter its conformation (Fig. 5d) as mechanical work (rotation) of the molecular motor assembly. Compared to a control framework, i.e., a non-motor variant with *meso*-α,β-di(4-pyridyl) glycol pillar, the moto-Py-TCPP(Pd) MOF showed a significant decrease in phosphorescence intensity (at 690 nm; Fig. 5e) with a shorter phosphorescence lifetime (ca 10 µs in moto-Py-TCPP(Pd) compared to 100 µs in non-motorized variant). Raman spectroscopic data were used to support the rotatory motion of pillar linkers, where a 530 nm irradiation changes the intensity of 1550 cm^−1^ peak corresponding to the thermal isomerisation^69^.

### Photoinduced charge transfer (PCT)

One of the most advantageous features of the PCT in MOF is its modularity achieved via its ability to systematically incorporate required complementary dyes or redox species in a controlled and periodic fashion. The pore size and geometry of the frameworks determine what can be incorporated within it and dictate if the incorporation can be performed de novo or through a post-synthesis process. The former technique is used to incorporate a non-structural entity trapped in a “ship in a bottle” fashion within the porous cages. Here, the guest molecule—being a bit larger than the largest pore window—remains “corked” inside the pore space with some degrees of restricted conformational and rotational freedoms. The encapsulated molecules can involve non-covalent interaction with the MOF surface. Larsen and co-workers encapsulated two porphyrin guests—tetrakis-(4-sulphonatophenyl)porphyrinato zinc(II) (T4SPP(Zn)) and tetrakis-(4-sulphonatophenyl)porphyrinato iron(III) (T4SPP(Fe))—into the cages of HKUST-1(Zn) MOF forming T4SPP(Zn)-T4SPP(Fe)@HKUST-1^70^. In these low-density porphyrin MOF composites, the PCT was observed from ^3^T4SPP(Zn)* to the T4SPP(Fe). This observation is in agreement with the reduction of the ^3^T4SPP(Zn) lifetime from 890 to 83 µs with an electron transfer rate *k*_CT_ ∼ 1.1 × 10^4^ s^−1^.

**Fig. 6:**
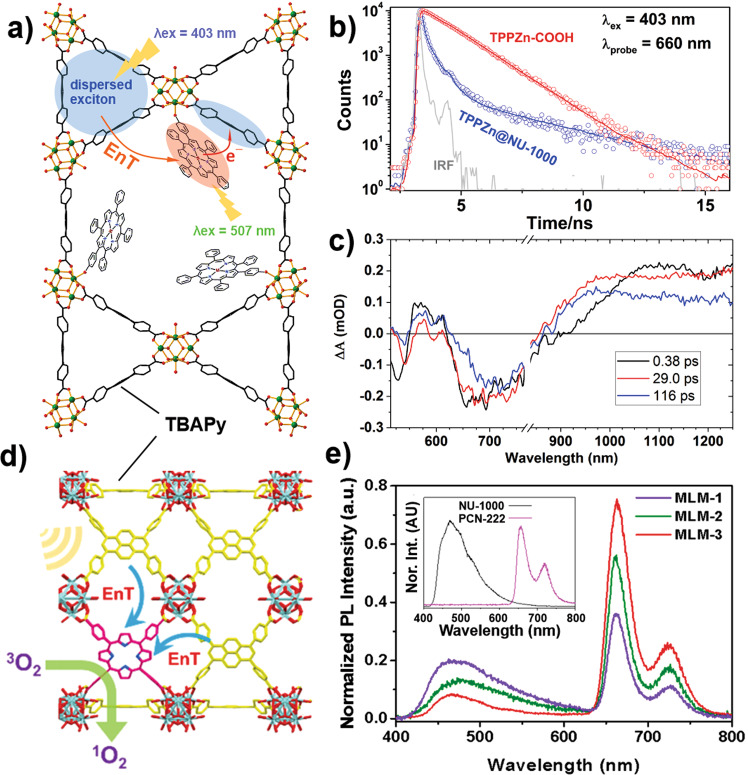
Photoinduced EnT and CT within low-density porphyrin MOFs. **a** Schematic representation of EnT and PCT in TPP(Zn) anchored NU-1000; **b** transient emission decay profiles of TPP(Zn)@NU-1000 and TPP(Zn); **c** representative fs-TA spectra of TPP(Zn)@NU-1000 under the excitation of 400 nm; **d** schematic rendering of EnT in TCPP(H_2_)-TBAPy MLM MOF; **e** representative fluorescence spectra of MLM MOFs with varying ratios of TCPP(H_2_) and TBAPy linkers (inset shows the fluorescence spectra of the pristine NU-1000 and PCN-222(H_2_) MOFs). **a**–**c** reprinted with permission from ref. ^71^; Copyright (2019), American Chemical Society. **d**, **e** reprinted with permission from ref. ^72^; Copyright (2017) American Chemical Society.

Li et al. constructed a low-density porphyrin framework system, where a carboxy-appended tetraphenylporphyrinato zinc(II) (TPP(Zn)) chromophore was post-synthetically installed at the Zr^IV^-oxo node within the one-dimensional pores of a TBAPy-MOF, NU-1000 (H_4_TBAPy = 1,3,6,8-tetrakis(*p*-benzoicacid) pyrene)^71^. The design of this TPP(Zn)@NU-1000 is based on the appropriate alignment of the respective ground- and excited-state redox potentials of these complementary components in a way that facilitates an efficient EnT from excited NU-1000* to TPP(Zn) forming TPP(Zn)* (Fig. 6a–c). The TBAPy assembly in the framework serves as the antenna to harvest photonic energy and efficiently deliver it to the adjacent TPP(Zn) anchored on the Zr^IV^-oxo node via FRET with *k*_EnT_ ≈ 4.7 × 10^11^ s^−1^ (*τ* = 2 ps). The excited-state oxidation potential of TPP(Zn)* is 0.54 eV higher than the ground-state reduction potential of NU-1000, hence a spontaneous PCT was observed from TPP(Zn)* to NU-1000 with *k*_CT_ = 1.2 × 10^10^ s^−1^. The PCT was probed via the solvent polarity-dependent time-resolved emission spectra: *k*_CT_ = 6.2 × 10^8^ s^−1^ and 1.2 × 10^10^ s^−1^ (i.e., *τ*_CT_ ~ 1.6 ns and 80 ps) were measured in 2-methyl tetrahydrofuran (MeTHF) and α,α,α-triflurotoluene (CF_3_Tol) solvents, respectively. The femtosecond transient absorption data collected for TPP(Zn)@NU-1000 (in CF_3_Tol solvent) captured the broad NIR transient signature of TPP(Zn)^•+^ at 976 nm as one of the PCT products. The global fitting of the transient dynamical data revealed a 283-ps charge recombination time constant. This work used the assembly of linkers with complementary optoelectronic properties to obtain more spectral coverage for light-harvesting applications. With a lower lowest-unoccupied molecular orbital energy level for the free-base variant TPP(H_2_), no significant PCT was observed; in this case, an efficient EnT of NU-1000* → TPP(H_2_) was expected. Indeed, in a separate study with a mixed (scrambled) linker MOF (MLM), Lee and co-workers have shown that to be the case. Modified NU-1000 MOF samples with varying concentrations of TCPP(H_2_) as a low-density linker were prepared via de novo syntheses from H_4_TCPP(H_2_) and H_4_TBAPy (Fig. 6d)^72^. Efficient EnT was observed in these MLMs from the pyrene to porphyrin: the emission spectra of the MLM highlight a large reduction in emission intensity at 470 nm (for pristine NU-1000*) and a corresponding increase in emission from the TCPP(H_2_) linker at 670 nm, compared to the emission profiles of pristine NU-1000 and PCN-222(H_2_) MOFs (Fig. 6e). The TCPP(H_2_)*, generated via such light-harvesting process, was exploited for efficient photochemical production of ^1^O_2_ through the formation of ^3^TCPP(H_2_)*.

## Electrochemical properties and charge transport processes in porphyrin MOFs

Considering MOFs as emerging compositions for energy conversion and storage applications, MOF-based working electrodes have become a central theme for various electrochemical developments. In this regard, MOFs can be ideal compositions as their pores ensure the substrate and electrolyte diffusion to access a large number of redox sites (per unit electrode area) compared to a monolayer-coated electrode. Moreover, for electrocatalysis and related developments, the framework assembly of redox-active species (e.g., TCPP(Fe), etc.) can suppress unproductive loss (commonly occurs via irreversible side reaction, thermal diffusion, or precipitation due to poor solubility) of intermediates formed during the progressive multielectron transformation of the resting catalyst assembly into the active form. The charge transport process in common metal–carboxylate MOFs is defined by site-to-site charge hopping that is coupled with counter ion diffusion (i.e., an asymmetric self-exchange)^73^. The site-to-site hopping process can be described by the Marcus theory^74,75^ for predictable improvements, which will be useful for electrocatalysis/photoelectrocatalysis^76–78^.

In biological systems, coordinatively saturated heme proteins constitute the redox cofactors of the cytochrome-based electron shuttles. In this regard, metalloporphyrins are electrochemically well-studied systems owing to their unique *d*-electron configurations relative to the porphyrin macrocycle core^79,80^. Morris and co-workers have prepared CoPIZA/FTO by growing the CoPIZA MOF from TCPP(Co^III^) linker and Co^II^-carboxylate node on the fluorine-doped tin oxide (FTO) electrode^81^. These films showed catalytic activity for carbon tetrachloride reduction. Interestingly, electrochemical impedance spectroscopic data collected for these MOF-modified electrodes revealed a conductivity of 3.62 × 10^−8^ S cm^−1^—a behavior that may have been augmented by the redox contribution from both the struts and Co nodes. Cyclic voltammogram (CV) of CoPIZA (@FTO) evinced two reversible cathodic peaks at −1.1 and −1.45 V (vs ferrocyanide) corresponding to TCPP(Co^III/II^) and TCPP(Co^II/I^), respectively. Interestingly, Co^III^ SBU shows similar redox potential values at −0.87 and −1.39 V corresponding to Co^III/II^ and Co^II/I^ redox processes, respectively. The current density and the cathodic–anodic peak separation of these redox events were found to be scan-rate dependent, and therefore, the charge transport rate (*k*_CT_) in this diffusion-limited process can be expressed in terms of the diffusion coefficient (*D*_hop_ ~ 7.55 × 10^–14^ cm^2^ s^−1^). Hod’s group reported tuning redox conductivity by engineering the defect site density with iron porphyrin ligands in a UiO-66 type MOF^82^. The Zr nodes with varying degrees of missing linker (and the corresponding void space) were used to install Fe-porphyrin (Hemin) molecules via SALI. These hemin-decorated UiO-66 MOFs were used to probe the hopping rate as a function of porphyrin loading (i.e., originally the defect density) that defines the variable (average) Fe–Fe distances. Transient electrochemical data revealed that the higher hemin-loaded (i.e., a shorter *d*_Fe-Fe_) samples manifest a low diffusion rate and the maximum diffusion rate was obtained for relatively lower hemin-loaded samples. Considering microporous UiO-66 system, a highly defective sample that led to high hemin loading allowed the decoration deep within MOF crystal, which seemed to impede the mass transport of the counterions. Whereas, in low-defect samples, the hemins mostly reside at the surface and therefore suppress the mass transport-related issues.

**Fig. 7:**
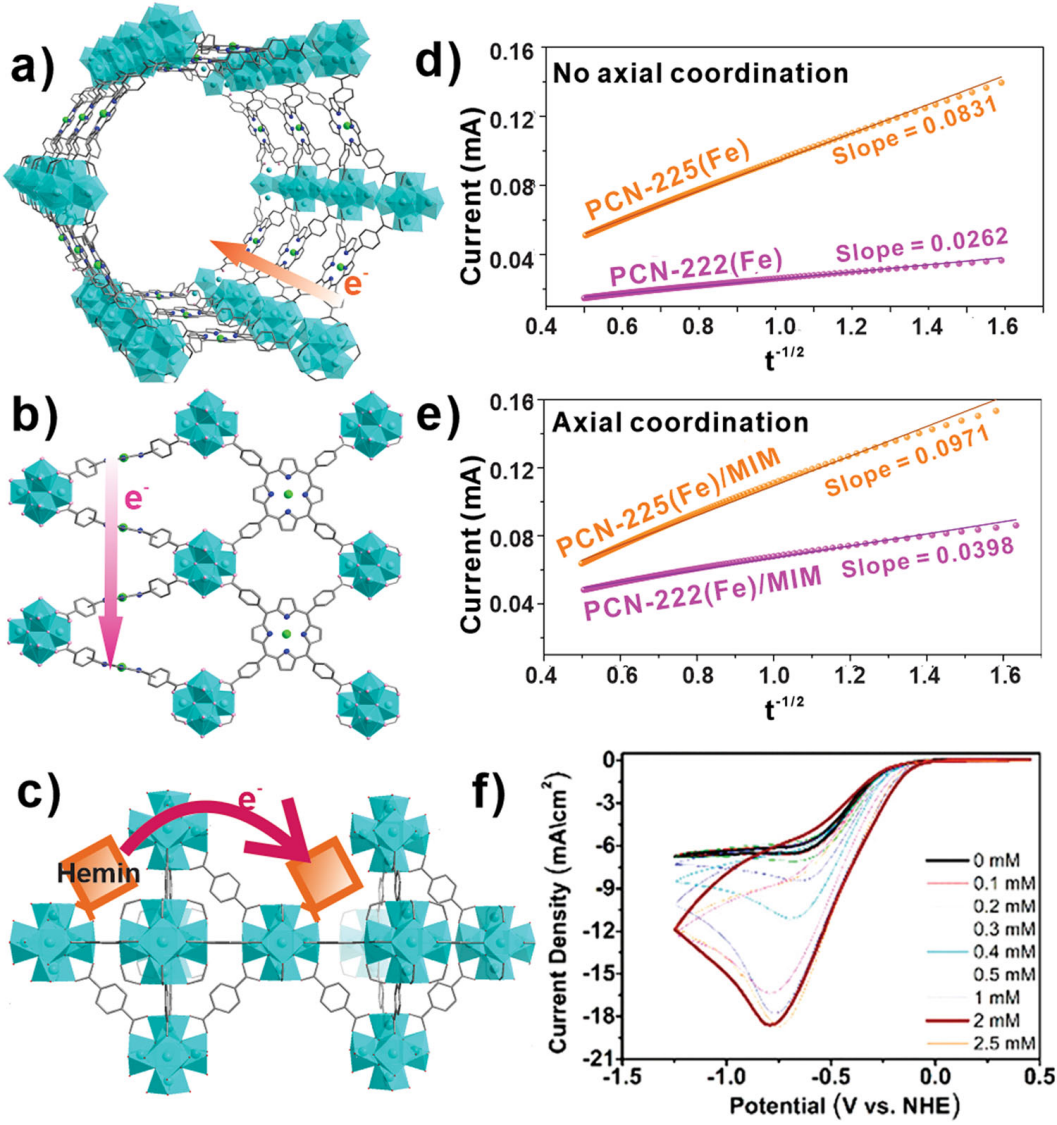
Charge hopping mediated transport processes in porphyrin MOF assemblies. Charge hopping within **a** PCN-222(Fe), **b** PCN-225(Fe) and their corresponding Cottrell plots **d**, **e** with and without MIM, respectively; **c** schematic representation of electron transfer in Hemin@UiO66 and **f** comparison of ORR performance as a function of MIM concentration. Figure panels **d**, **e** reprinted with permission from ref. ^83^; Copyright (2019) American Chemical Society. Figure panel **f** reprinted with permission from ref. ^84^; Licensed by ACS, link: 10.1021/jacs.9b11355 and further permissions related to the material excerpted should be directed to the ACS.

We know that the five-coordinated Fe^III^ and Fe^II^ sites exert domed structures relative to the macrocyclic plane of the porphyrin as the metal ions in their high spin (HS) state possess a larger size than the ligand cavity. Therefore, a chloro/solvent-coordinated TCPP(Fe^III^Cl) ⟷ TCPP(Fe^II^) will involve a HS Fe^III^ (S = 5/2) to HS Fe^II^ (S = 2; or an intermediate spin S = 1 in a four-coordinated environment) transition that would require higher reorganization energy. In contrast, a six-coordinated TCPP(Fe^III^L_2_) ⟷ TCPP(Fe^II^L_2_) transformation would involve a planar TCPP(Fe^III/II^) in their low spin (LS) state requiring less internal reorganization energy. Using 1-methylimidazole (MIM) as the axial ligand with four structurally different TCPP(Fe^III^)-derived Zr-MOFs, PCN-222(Fe) (*csq*), PCN-225(Fe) (*sqc*), NU-902(Fe) (*scu*), and in MOF-525(Fe) (*ftw*), Deria group has studied the redox hopping as a function of framework topology and probed the impact of the spin state of the central metal ions at the porphyrin^83^. The CV plots revealed a TCPP(Fe^III/II^) redox event at −0.5 V (vs Ag/AgCl), which cathodically shifted in the presence of MIM (TCPP(Fe^III/II^) redox event at −0.7 V). This potential shift reflects a sterically demanding access of the iron sites by the axial ligands and is therefore more pronounced in the PCN-225(Fe) with the smallest pore size. Owing to smaller reorganization energy, coordination of two axial MIM ligands enhanced the charge transport that increased the current density. The diffusion coefficients (*D*_hop_), representing the site–site charge-carrier hopping (coupled with the counterions), were obtained from the slope of the Cottrell plot (Fig. 7d, e) and then converted to hopping rates (*k*_hop_) using the center–center distance (*d*_Fe-Fe_). For the larger pore PCN-222(Fe), a smaller *k*_hop_ value (64 s^−1^), and for the narrow-pore PCN-225(Fe), a large *k*_hop_ value (890 s^−1^) was determined. In the presence of MIM, however, the larger pore MOFs evinced ~3–4 times improvement in *k*_hop_ where the smaller pores could not accommodate two axial MIM and evinced a nominal improvement. The supporting Raman spectral data revealed that the large pore PCN-222(Fe^III^) and smaller pore PCN-225(Fe^III^) exist mostly in LS and HS state, respectively, in the presence of MIM. Thus, NU-902 with intermediate pore size manifested the highest *k*_hop_ value 1480 s^−1^ in the presence of MIM. These results suggest an interesting possibility for electrocatalysis: a five-coordinated Por(Fe^III/II^) will have better charge-carrier conductivity upon substrate binding. In a contemporary electrocatalytic oxygen reduction reaction (ORR) work, Hod and co-workers have found that a large improvement in the hopping rate in the Hemin@UIO-66 (vide supra) can be achieved in the presence of MIM (rate 3858 s^−1^)^84^ and established that such process can be beneficial, leading to an improved electrocatalytic ORR at the hemin(Fe^III/II^) redox event. Much like the natural heme in peroxidases (e.g., hydrogen peroxidase) where an imidazole (histidine) binding stabilizes a LS complex triggering the O-O bond activation (and breaking), the Hemin@UIO-66 not only entailed an improved charge hopping rate in the presence of MIM (axial coordination) but also increased the ORR rate (Fig. 7c, f). The catalytic current increased from −6.4 to −18.58 mA cm^−2^ (~300% increment) for 0 and 2 mM MIM, respectively.

The incorporation of redox-active guests within the MOF pores has been explored as a viable strategy to improve the conductive behavior of the frameworks. Even though these redox-active guest species can provide mid-gap states, the actual process may differ mostly by the potential energy gap and the nature of the host–guest interaction (i.e., forming discrete mid-gap bands localized over host/guest or a delocalized one). Among these, ferrocene, fullerenes, and 7,7,8,8-tetracyanoquinodimethane (TCNQ) have been incorporated and studied. Ferrocene, incorporated within MOFs, behaves like an electronically isolated entity^85^; neither Fc/Fc^+^ nor the host/host^+^ potential was found to be significantly perturbed by such an assembly—even though the short Fc-linker distance facilitates fast PCT^86^. TCNQ, on the contrary, has been used to wire the MOF building units via coordination at the open sites and facilitates electronic mixing in the ground state^78^. The conductivity of a 2D Cu-MOFs, constructed from 5,10,15,20-tetra-4-pyridyl-porphyrin (TPyP(Cu); in situ metallation during MOF synthesis, was seen to improve by 1000-fold upon TCNQ doping (*σ* = 10^–4^ S m^−1^) at room temperature^87^.

**Fig. 8:**
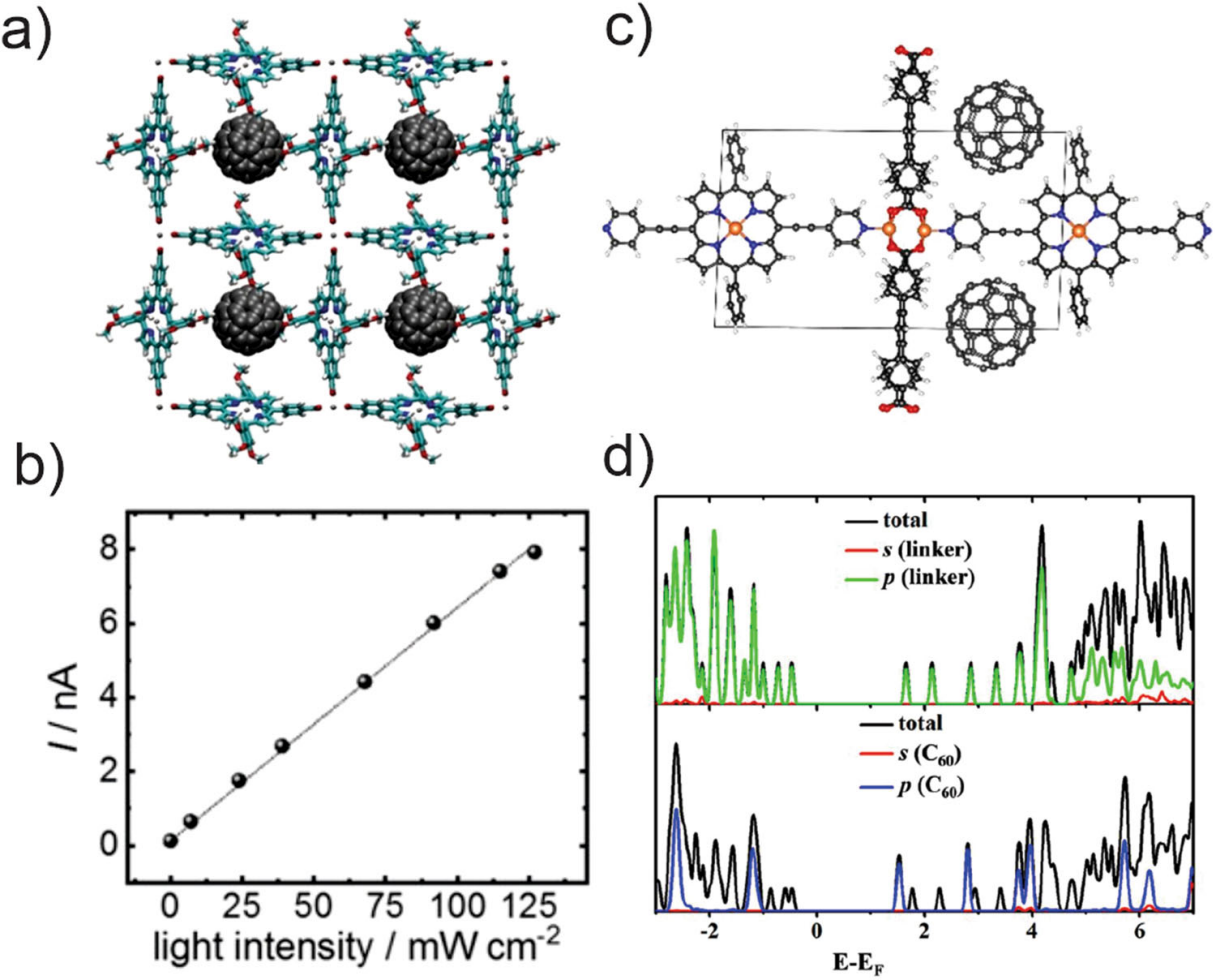
Fullerene infused porphyrin MOFs and their electronic properties. Structure of **a** C_60_@TMPCP(Zn) MOF and its **b** photocurrent at different intensities of 455 nm irradiation; **c** structure of C_60_@DA-MOF and **d** computed density of states. Figure panels **a**, **b** reprinted with permission from ref. ^88^; Copyright (2019) Wiley-VCH. Figure panels **c**, **d** reprinted with permission from ref. ^90^; Copyright (2020) American Chemical Society.

Fullerene–porphyrin had been well-established molecular electron donor–acceptor dyad systems (fullerene being the electron-deficient entity). The integration of fullerene or its derivatives into the pores can be done during the MOF assembly from porphyrin linkers; this strategy is useful for microporous or closely packed layered MOFs. For example, Liu and co-workers reported a significantly enhanced photoconductivity (Fig. 8a, b) in a fullerene-infiltrated MOF consisting of 5,15-bis-(3,4,5-trimethoxyphenyl)-10,20-bis-(4-carboxyphenyl) porphyrinato zinc(II) (TMPCP(Zn)). Upon Soret band excitation (455 nm), the fullerene–MOF hybrid entailed a photoconductivity of 1.3 × 10^–7^ S m^−1^—a four order of magnitude improvement compared to its dark conductivity (1.5 × 10^–11^ S m^−1^)^88^. The linear correlation between the photocurrent and the intensity of incident light was used to establish a single photon-mediated PCT process. The computational results suggested that a significant electronic coupling through the highest occupied valence orbitals of porphyrin linkers, compared to other intermolecular pairs in this MOF, could be a key factor behind the hole transport path along the stacking direction of porphyrin linkers (i.e., *z*-axis). This work showed that PCT between porphyrin–fullerene dyad, established within this MOF samples, leads to a high concentration of charge carriers.

Fullerenes can also be infiltrated within 1D MOF pores post-synthetically via dispersive interactions with the aromatic organic linkers, where a linker–fullerene interaction can increase the conductivity. Based on the experimental work by the Hupp group on fullerene (C_60_)-packed NU-901 (a pyrene MOF) that boosted the MOF conductivity from 10^–14^ to 10^–3^ S cm^−1^ ^89^, Cramer and co-workers computationally investigated C_60_@F-MOF and C_60_@DA-MOF^90^. Their results suggested that the fullerene-infused hybrids entail a lower bandgap by 0.36 and 0.16 eV, respectively. Partial density of state analysis evinced a porphyrin (linker)-derived highest occupied crystal orbital (HOCO) with C_60_-centered lowest unoccupied crystal orbitals (LUCO; Fig. 8c, d). Such interactions were expected to improve the charge-carrier transport, possibly by lowering the activation energy. In related work, Cramer’s group further showed that fullerene (as well as metal biscarbollides, M^IV^(C_2_B_9_H_11_)_2_; M = Ni, Pd, Pt) incorporation within the triangular pore of the PCN-222(H_2_) (and PCN-222(Zn)) can cause a reduced HOCO–LUCO gap. Such hybrids hold promise for improving the PCT as well as charge transport within MOFs^91^, where the mesopores can provide required chemical accessibility.

## Applications

With these basic photophysical and electrochemical understandings, numerous porphyrin MOFs have been designed and explored for the development of organic photovoltaics (OPVs)^32^ as well as electrocatalytic^92,93^ and photoelctrocatlytic^94^ transformations like CO_2_ and proton reductions. For example, Wöll and co-workers have shown that the closely packed layers of SURMOFs can be exploited for OPV applications^32^. Based on time-dependent density functional theory computations, a library of ditopic porphyrin linkers with different *meso* and *β* substitutions were created with their tunable Soret and *Q*-band absorptions. From this library, three candidate porphyrin molecules with superior and desired absorption spectral features were chosen for SURMOF fabrication. The fabrication of a “tri-depth” SURMOF with the three different porphyrin linkers was expected to be a route to cover the absorption from violet to NIR region of solar spectra. In a different direction, Hupp et al. reported MOF-525(Fe)-modified electrode providing a large areal concentration of Por(Fe) that can be sequentially transformed to active Por(Fe^I/0^) for CO_2_ reduction reaction (CRR)^92^. Hod and co-workers studied electron transfer kinetics on the surface of MOF-525(H_2_) photoelectrode^94^ by introducing an electron or hole acceptor in the electrolyte. It was found that these hole/electron acceptors can successfully define the polarity of photoelectrode; for example, introducing triethanolamine (a hole quencher) resulted in a photo-anodic response, whereas water (an electron acceptor) entailed a photo-cathodic response to produce H_2_. Likewise, numerous photocatalytic CRR (and H_2_ evolution) have been known with porphyrin-based MOFs^95–99^. Visible light-driven CRR with Por(Fe)-MOF has been achieved via Ir(ppy)_3_-photosensitizer (*E*^0^(Ir(ppy)_3_^+^/Ir(ppy)_3_*) ≈ −1.73 V is suitable for activating *E*^0^(Fe^I^/Fe^0^) = −1.58 V)^100^. Alternatively, as reported by Ma and co-workers, a Ti^IV^-modified Zr^IV^-oxo node in MOF-525(H_2_) can be used^101^ to achieve photo-reduced Ti^III^@Zr^IV^-oxo node driving CRR to methane and CO in the aqueous media (water being the hole quencher for TCPP(H_2_)^+^ regeneration). Likewise, EnT within porphyrin-based MOFs was exploited for photodynamic therapy (PDT) to treat tumor cells^102^. A MOF-based system can be beneficial for PDT as (a) precisely aligned porphyrin transition dipoles in MOF can suppress many wasteful self-quenching processes that hamper efficient harvest of the triplet excited states, (b) high concentration of porphyrins can provide better (photo) catalytic performance to produce ^1^O_2_, and (c) pore-facilitated diffusion of molecular oxygen along with exciton migration can augment ^1^O_2_ production. Lin’s group constructed a nano-sized Hf-MOF DBU-UiO from 5,15-di(p-benzoicacid)porphyrin (H_2_DBP(H_2_)) linker^103^, where the Hf (presumably with large spin–orbit coupling) was proposed to facilitate S_1_ → T_1_ ISC, which led to the improvement in photosensitized ^1^O_2_ production for PDT. Among other applications, MOF-assembled TCPP(Mn) were found superior for ^1^O_2_ generation by degrading hydrogen peroxide under light compared to a solvent-dissolved TCPP(Mn) or solid aggregates. The electron transfer and charge transport properties discussed above will essentially provide suitable platforms for more such electrocatalytic, photocatalytic, or photoelectrocatalytic developments.

## Outlook

The well-defined arrangements of porphyrins in ordered frameworks give rise to unique optoelectronic properties that are difficult to achieve in solution-dissolved states. Interchromophoric interactions, among the neighboring porphyrins or between two complementary chromophores (one being a porphyrin), can be advantageous for efficient EnT and photoinduced charge separation processes. Topological modulation has been shown to play a big role in determining the inter-porphyrin distance and/or the ability to install a desired secondary component (e.g., electronically different porphyrin, fullerene derivatives, and so on) to facilitate both exciton and charge migration. Metal nodes with large electronic bandgaps (e.g., Zr^IV^-oxo, Zn^II^-carboxy, etc.) have made it possible to study the evolution of unique linker-derived excitonic features and enabled probing their dynamics. However, these could be detrimental in ground-state electronic modulation, as charge carriers will have to hop across these electronic nodes. On the contrary, nodes constructed from redox-active metal ions (like Co^III^, Fe^III^, Cu^II^, etc.) can enable mixing with the redox-active metalloporphyrins or provide mid-gap states to contribute to ground-state electronic features, including improved charge conductivity.

These fundamental results showed us tremendous possibilities that porphyrin-based MOFs offer for future developments. In this regard, extensive photophysical developments defining excitonic properties and dynamics will help us to delineate ways to vectorially drive excitons (to the charge separation site or a catalytic site). Likewise, optimal electronic control is required to drive the charge specifically and directionally. Eventually, the goal will be to maximize the quantum yield of usable redox equivalents (i.e., charges with appropriate potential)—such systems will feature required electronic properties and (photo)conductivities to deliver them to the catalytic sites (or electrode) for photocatalytic and photoelectrocatalytic energy conversion, OPV, and analytical developments. Alternatively, electronic modulation of the porphyrins and their assemblies will feature novel compositions with high charge mobility for electrocatalytic developments; it will not be far-fetched to think of porous porphyrin MOFs as charge storage systems—given their high surface areas. Nevertheless, the underlying pace of such developments will critically depend on new liker design leading to desired MOF structures. Theory-based works may come into the picture in identifying and mapping such molecular and framework structures. Overall, the future of this exciting interdisciplinary field will depend on how we successfully merge decade-old rich photochemistry and electrochemistry with highly modular and adaptive MOF chemistry. For example, porphyrins with lower electronic symmetry or those with high triplet yields (and long lifetime) could be exploited to generate appropriate MOF structures that can be deposited on various electrodes—like the SURMOFs for photo or electrochemical processes. This article highlights and marks only the onset of a plethora of new developments in the near future.
